# Supplementary material for: Catastrophic famine in Gaza: Unprecedented levels of hunger post-October 7th. A real population-based study from the Gaza Strip
Source: PLoS One. 2025 May 28;20(5):e0309854. doi: 10.1371/journal.pone.0309854 (PMC12118885; doi:10.1371/journal.pone.0309854)
Supplement: S1 Table — (DOCX) [file pone.0309854.s001.docx]

**Supplementary Table 1:** Correlation between the three food security/hunger assessment tools and the sociodemographic characteristics of the study households in the Gaza Strip.

| **Sociodemographic characteristics** | | **HHS total score** | **HFSSM total score** | **HFIAS total score** |
| --- | --- | --- | --- | --- |
| Number of displacement times | Pearson Correlation | **0.161**** | **0.077**** | 0.036 |
|  | P-value | **0.000** | **0.008** | 0.208 |
| Age | Pearson Correlation | **0.139**** | **0.090**** | **0.098**** |
|  | P-value | **0.0001** | **0.002** | **0.001** |
| Age of partner | Pearson Correlation | **0.132**** | 0.046 | **0.106**** |
|  | P-value | **0.0001** | 0.112 | **0.0001** |
| Number of family members | Pearson Correlation | 0.052 | 0.023 | 0.005 |
|  | P-value | 0.069 | 0.420 | 0.864 |
| Number of dependent children | Pearson Correlation | 0.054 | -0.001 | 0.022 |
|  | P-value | 0.063 | 0.985 | 0.435 |
| How many children have symptoms | Pearson Correlation | **0.150**** | **0.092**** | **0.071*** |
|  | P-value | **0.0001** | **0.001** | **0.014** |
| How many children died because of starvation | Pearson Correlation | 0.036 | **0.069*** | 0.036 |
|  | P-value | 0.209 | **0.017*** | 0.215 |
| Weight before war | Pearson Correlation | -0.052 | -0.014 | -0.009 |
|  | P-value | 0.073 | 0.616 | 0.767 |
| How many kilograms have you lost in weight since the war started | Pearson Correlation | 0.039 | -0.048 | **-0.082**** |
|  | P-value | 0.180 | 0.098 | **0.004** |
| Current calculated weight | Pearson Correlation | **-.075**** | 0.011 | 0.037 |
|  | P-value | **0.009** | 0.691 | 0.203 |
| BMI current | Pearson Correlation | **-.097**** | -0.044 | 0.006 |
|  | P-value | **0.001** | 0.128 | 0.834 |
| BMI before the war | Pearson Correlation | **-0.071*** | **-0.068*** | -0.036 |
|  | P-value | **0.013** | **0.018** | 0.206 |
