# Supplementary material for: Catastrophic famine in Gaza: Unprecedented levels of hunger post-October 7th. A real population-based study from the Gaza Strip
Source: PLoS One. 2025 May 28;20(5):e0309854. doi: 10.1371/journal.pone.0309854 (PMC12118885; doi:10.1371/journal.pone.0309854)
Supplement: S2 Table — (DOCX) [file pone.0309854.s002.docx]

**Supplementary Table 2:** HHS vs. sociodemographic cross-tabulation and chi-square analysis.

| **Variable** | | **Little or no household hunger** | **Moderate household hunger** | **Severe household hunger** | **P-value** |  |
| --- | --- | --- | --- | --- | --- | --- |
| **City** | Northern  (Gaza City and North Gaza) | 23 (16.3) | 130 (26.4) | 178 (31.0) | **˂ 0.001*** | |
|  | Middle  (Deir Al Balah) | 48 (34.0) | 156 (31.6) | 91 (15.8) |  |  |
|  | Southern (Khan Younis and Rafah) | 70 (49.7) | 207 (42.0) | 306 (53.2) |  |  |
| **Sex** | Male | 65 (46.1) | 227 (46.1) | 270 (46.9) | 0.967 | |
|  | Female | 76 (53.9) | 265 (53.9) | 306 (53.1) |  |  |
| **Responsible for the family** | Man | 131 (92.9) | 462 (93.9) | 494 (85.8) | **˂ 0.001*** | |
|  | Woman | 10 (7.1) | 30 (6.1) | 82 (14.2) |  |  |
| **Economic status before the war** | Low | 24 (17.0) | 178 (36.2) | 210 (36.5) | **˂ 0.001*** | |
|  | Medium | 96 (68.1) | 288 (58.5) | 314 (54.5) |  |  |
|  | High | 21 (14.9) | 26 (5.3) | 52 (9.0) |  |  |
| **Starvation symptoms appear** | Yes | 86 (61.0) | 402 (81.7) | 525 (91.1) | **˂ 0.001*** | |
|  | No | 55 (39.0) | 90 (18.3) | 51 (8.9) |  |  |
| **Did any child die because of starvation** | Yes | 0 (0.0) | 2 (0.4) | 3 (0.5) | 0.688 | |
|  | No | 141 (100.0) | 490 (99.6) | 573 (99.5) |  |  |
| **Marital status** | Married | 136 (96.5) | 471 (95.7) | 554 (96.2) | 0.207 | |
|  | Widowed | 1 (0.7) | 16 (3.3) | 16 (2.8) |  |  |
|  | Divorced | 4 (2.8) | 5 (1.0) | 6 (1.0) |  |  |
| **Partner working** | Yes | 75 (53.2) | 210 (42.7) | 172 (29.9) | **˂ 0.001*** | |
|  | No | 66 (46.8) | 282 (57.3) | 404 (70.1) |  |  |
| **The educational level of the partner** | Primary | 2 (1.4) | 4 (0.8) | 29 (5.0) | **0.003*** | |
|  | Elementary | 13 (9.2) | 69 (14.0) | 61 (10.6) |  |  |
|  | Secondary | 43 (30.5) | 166 (33.7) | 175 (30.4) |  |  |
|  | BSc | 79 (56.0) | 233 (47.4) | 281 (48.8) |  |  |
|  | MSc | 3 (2.1) | 16 (3.3) | 27 (4.7) |  |  |
|  | PhD | 1 (0.7) | 4 (0.8) | 3 (0.5) |  |  |
| **House status** | Total destruction | 57 (40.4) | 279 (56.7) | 321 (55.7) | **˂ 0.001*** | |
|  | Partial destruction | 53 (37.6) | 139 (28.3) | 180 (31.3) |  |  |
|  | No Destruction | 21 (14.9) | 45 (9.1) | 32 (5.6) |  |  |
|  | I do not know | 10 (7.1) | 29 (5.9) | 43 (7.5) |  |  |
| **Address before the war** | City house/Home | 102 (72.3) | 425 (86.3) | 454 (78.8) | **0.001*** | |
|  | Camp | 39 (27.7) | 67 (13.6) | 122 (21.2) |  |  |
| **Current address** | Tent | 72 (51.1) | 250 (50.8) | 385 (66.8) | **˂ 0.001*** | |
|  | Home | 47 (33.3) | 91 (18.5) | 70 (12.2) |  |  |
|  | School | 22 (15.6) | 151 (30.7) | 121 (21.0) |  |  |
| **Do you receive any help from a relief organization** | No | 8 (5.7) | 107 (21.7) | 120 (20.8) | **˂ 0.001*** | |
|  | Regularly | 6 (4.3) | 9 (1.8) | 15 (2.6) |  |  |
|  | Intermittent | 127 (90.1) | 376 (76.4) | 441 (76.6) |  |  |
