# Supplementary material for: Catastrophic famine in Gaza: Unprecedented levels of hunger post-October 7th. A real population-based study from the Gaza Strip
Source: PLoS One. 2025 May 28;20(5):e0309854. doi: 10.1371/journal.pone.0309854 (PMC12118885; doi:10.1371/journal.pone.0309854)
Supplement: S3 Table — (DOCX) [file pone.0309854.s003.docx]

**Supplementary Table 3:** Predictors of food insecurity and hunger.

| **Variable** | | **OR** | **95% CI** | **P-value** |
| --- | --- | --- | --- | --- |
| **City** | Northern (reference)  (Gaza City and North Gaza) | - | - | - |
|  | Middle  (Deir Al Balah) | 2.771 | 1.273; 6.030 | **0.010*** |
|  | Southern  (Khan Younis and Rafah) | 1.930 | 1.076; 3.461 | **0.027*** |
| **Sex** | Male (reference) |  |  |  |
|  | Female | 0.073 | 0.048; 0.373 | **0.011*** |
| **Responsible for the family** | Man | - | - | - |
|  | Woman | 0.144 | 0.422; 0.956 | **˂ 0.001*** |
| **Economic status before the war** | Low (reference) | - | - | - |
|  | Medium | 1.687 | 0.915; 3.113 | 0.094 |
|  | High | 2.989 | 1.077; 8.298 | **0.036*** |
| **Starvation symptoms appear** | Yes (reference) | - | - | - |
|  | No | 0.187 | -0.948; -0.515 | **˂ 0.001*** |
| **Did any child die because of starvation?** | Yes (reference) | - | - | - |
|  | No | 0.047 | -2.321; 0.208 | 0.101 |
| **Marital status** | Married (reference) | - | - | - |
|  | Widowed | 2.004 | 0.381; 10.556 | 0.412 |
|  | Divorced | 0.802 | 0.111; 5.795 | 0.827 |
| **Partner working** | Yes (reference) | - | - | - |
|  | No | 0.148 | 0.273; 0.605 | **˂ 0.001*** |
| **The educational level of the partner** | Primary (reference) | - | - | - |
|  | Elementary | 2.429 | 0.678; 8.701 | 0.173 |
|  | Secondary | 1.160 | 0.393; 3.427 | 0.788 |
|  | BSc | 2.532 | 0.866; 7.405 | 0.090 |
|  | MSc | 0.286 | 0.046; 7.405 | 0.178 |
|  | PhD | 2.044 | 0.0; 0.0 | 1.000 |
| **House status** | Total destruction (reference) | - | - | - |
|  | Partial destruction | 1.187 | 0.658; 2.145 | 0.569 |
|  | No Destruction | 0.704 | 0.255; 1.939 | 0.497 |
|  | I do not know | 0.679 | 0.219; 2.131 | 0.507 |
| **Address before the war** | City (reference) | - | - | - |
|  | Home | 0.658 | 0.144; 3.017 | 0.590 |
|  | Camp | 4.167 | 1.368; 12.691 | **0.012*** |
| **Current address** | Tent (reference) | - | - | - |
|  | Home | 1.040 | 0.503; 2.153 | 0.916 |
|  | School | 0.815 | 0.433; 1.532 | 0.525 |
| **Do you receive any help from a relief organization** | No (reference) | - | - | - |
|  | Regularly | 1.232 | 0.310; 4.898 | 0.767 |
|  | Intermittent | 1.043 | 0.556; 1.959 | 0.895 |
