# Supplementary material for: Catastrophic famine in Gaza: Unprecedented levels of hunger post-October 7th. A real population-based study from the Gaza Strip
Source: PLoS One. 2025 May 28;20(5):e0309854. doi: 10.1371/journal.pone.0309854 (PMC12118885; doi:10.1371/journal.pone.0309854)
Supplement: Attachment — Submitted filename: Feedback PLOS ONE.pdf [file pone.0309854.s006.pdf]

Feedback PLOS ONE

General Feedback

The manuscript titled **"Catastrophic Famine in Gaza: Unprecedented Levels of Hunger Post-October 7th. A Real Population-Based Study from the Gaza Strip"** provides critical insights into hunger and food insecurity in Gaza amidst a military conflict. The study addresses a timely and significant humanitarian issue, offering valuable data that highlights the severity of the crisis and informs potential policy and humanitarian interventions.

The use of robust tools such as the HFSSM, HFIAS, and HHS to measure food insecurity enhances the credibility and rigor of the findings. While the manuscript is generally well-structured, some sections are repetitive, and the language could benefit from further refinement to improve readability and align with academic standards.

The discussion effectively aligns to inform policymakers. However, it would benefit from more actionable recommendations and detailed strategies to address the crisis. Overall, the study has strong potential for publication following minor revisions.

Specific Comments

Abstract

- The abstract provides a concise summary but could better emphasize key findings and their implications.
- Revise the sentence on informing policymakers (lines 19–22) to focus on actionable insights rather than general aims.

Introduction

- While the introduction offers comprehensive background information, it could be more concise, especially when discussing the historical context of food insecurity in Gaza.
- Definitions of key terms like hunger, food insecurity, and famine (lines 76–94) are helpful but should be condensed to improve clarity and avoid redundancy.

Methods

- Details on inclusion criteria (lines 141–145) and the translation process (lines 152–154) should be expanded to enhance the reliability and validity of the methods.
- Elaborate on the training process for data collectors (lines 131–135) to strengthen the methodological rigor.

Results

- The results are detailed, but incorporating visual aids such as graphs or charts would enhance clarity and engagement, particularly for shifts in BMI and hunger prevalence.
- The categorization and impact of economic status on food insecurity (line 162) should be further elaborated.
- Provide clearer categorization and contextual analysis of displacement data (line 130).

Discussion

- The discussion is thorough but occasionally redundant. Focus more on how the findings can directly inform policy and humanitarian interventions.
- The study limitations (lines 615–637) are well-addressed but should also include potential biases related to self-reported data.

Conclusion

- The conclusion is consistent with the findings but introduces new elements, such as the cessation of hostilities (line 650), which should be integrated earlier in the discussion.

References

- Ensure consistency and accuracy in citations, particularly for references such as ICRC protocols (lines 40–41).

Figures and Tables

- Incorporate visual representations for key findings, such as weight loss and hunger severity, to improve the manuscript impact (e.g., BMI changes on Page 14).

Page-Specific Comments

- **Page 5, Line 62**: Incorporate more recent reports on hunger to provide up-to-date context.
- **Page 10, Line 162**: Clarify how economic status categories were defined and determined.
- **Page 14, Table 4**: Simplify the table and key trends in the narrative.
- **Page 18, Line 453**: Include comparisons with similar studies on food insecurity in other conflict zones to contextualize the findings.
