# Supplementary material for: Catastrophic famine in Gaza: Unprecedented levels of hunger post-October 7th. A real population-based study from the Gaza Strip
Source: PLoS One. 2025 May 28;20(5):e0309854. doi: 10.1371/journal.pone.0309854 (PMC12118885; doi:10.1371/journal.pone.0309854)
Supplement: Attachment — Submitted filename: Faris_Plosone.docx [file pone.0309854.s004.docx]

**Research Design and Rationale:**

1. **Clarity of Research Objectives**:
   - The abstract clearly states the aim to inform policymakers and humanitarian organizations, but could you clarify how the findings are specifically intended to inform policy or interventions in Gaza? Are there any particular groups or strategies you envision targeting with the findings?
2. **Justification of Study Location**:
   - Why did you choose to conduct this study in Gaza specifically during this period of conflict? Could you elaborate on how the timing of the study (May to July 2024) may have influenced the findings, given that the situation in Gaza has been highly dynamic?
3. **Previous Literature**:
   - How does your study compare to existing research on food insecurity in conflict zones, especially in Gaza? Are there any notable discrepancies with previous studies that report lower levels of food insecurity, and how do you explain these differences?

**Sampling and Data Collection:**

4. **Sampling Strategy**:
   - The study surveyed 1209 households out of an initial 1503. Could you provide more details about the reasons behind excluding 294 households? Was this due to missing data, or were there other factors that may have affected the final sample?
5. **Demographic Representation**:
   - You mention the mean age of participants is 38 years, with 53.5% being female. Were these demographics reflective of the overall Gaza population? Did you find any significant gender or age-based differences in food insecurity and hunger levels?
6. **Data Collection in a Conflict Zone**:
   - Given the ongoing conflict, were there any particular challenges or limitations in collecting data in Gaza? For example, how did the security situation impact the study’s logistics or participants' willingness to engage?
7. **Self-Reported Anthropometric Data**:
   - You collected self-reported anthropometric data, such as weight changes. How confident are you in the accuracy of these self-reports, considering the stress and trauma participants may be experiencing? Please insert this as limitations.

**Interpretation of Results:**

12. **Hunger and Weight Loss**:

- The average weight loss (from 74.6 kg to 64.1 kg) is striking. What do you believe are the key contributing factors to this weight loss beyond food insecurity, such as stress, healthcare access, or psychological impacts?

13. **Statistical Impact of Confounding Variables**:

- The abstract mentions that factors like displacement, age, economic status, and education significantly impacted hunger severity. Could you provide more details on the extent to which these variables influenced your findings? Were there any surprising or unexpected relationships?

**Policy and Humanitarian Implications:**

14. **Recommendations for Action**:

- Based on your findings, what specific recommendations would you propose to humanitarian organizations operating in Gaza to mitigate hunger and improve food security? Are there any quick-win interventions that could be implemented in the short term?

15. **Long-term Policy Recommendations**:

- Beyond immediate relief efforts, what longer-term policy changes do you think are necessary to address food insecurity in Gaza, particularly considering the ongoing conflict and blockade? Are there any impact on SDGs? Can you please indicate the importance of your study that shows the useless of international humanitarian rights and the SDGs.
