# Supplementary material for: Catastrophic famine in Gaza: Unprecedented levels of hunger post-October 7th. A real population-based study from the Gaza Strip
Source: PLoS One. 2025 May 28;20(5):e0309854. doi: 10.1371/journal.pone.0309854 (PMC12118885; doi:10.1371/journal.pone.0309854)
Supplement: Attachment — Submitted filename: PLOSONE Gaza Hunger Responses to Reviewers 25112024.docx [file pone.0309854.s007.docx]

**Responses to Reviewers’ Comments to the Author**

Dear Editor,

PLOSONE

I am writing to submit our rebuttal and authors' response to the reviewers' comments regarding our manuscript, “Catastrophic Hinger in Gaza: Unprecedented Levels of Hunger Post-October 7th. A Real Population-Based Study from the Gaza Strip,” which we submitted on PLOSONE. We sincerely appreciate the reviewers' insights and constructive feedback, which have led to significant improvements in our work. In our response, we have addressed each of the reviewers' comments point-by-point and provided clarifications and modifications to enhance the clarity and robustness of our manuscript. These revisions have strengthened our arguments and contributed to a more comprehensive understanding of our findings.

We are grateful for the opportunity to address these comments and hope our revisions meet the reviewers' expectations.

I appreciate your consideration, and we look forward to your positive response.

Best regards,

Faris, ME

Corresponding author

**Reviewer #1:**

**Comment:** This paper is an important contribution to documenting Israel's genocide against Palestinians. While I appreciate the authors' attempts to use "neutral" language to describe and document the levels of food insecurity and hunger currently experienced by Palestinians, it is critical that the authors consider the connections between the hunger and famine being experienced in Gaza and the concept of starvation as a weapon of a genocidal war being perpetrated by Israel.

**Response:** Thanks for admiring our work, glad to hear. The use of Israeli forces for food and hunger as a weapon in their battle against the Palestinians has been documented and reported in our work; see lines 401-409.

**Comment:** I would therefore like to recommend that the authors consider the report produced by Human Rights Watch on this matter: <https://www.hrw.org/news/2023/12/18/israel-starvation-used-weapon-war-gaza>. The report asserts that (1) The Israeli government is using starvation of civilians as a method of warfare in the Gaza Strip, which is a war crime, and (2) Israeli officials have made public statements expressing their aim to deprive civilians in Gaza of food, water, and fuel – statements reflected in Israeli forces’ military operations.

**Response:** Thanks, add as suggested, please see lines 462-468.

**Comment:** On a personal note, as an author who has struggled to publish papers in peer-reviewed journals because reviewers tend to question the neutrality and objectivity of my analysis of Israel's genocidal tactics, I completely understand your decision and approach to this paper. Consequently, my comments above should be considered a recommendation rather than a prerequisite for publication.

**Response:** Thank you for your understanding. It is appreciated and means a lot to the authors. The authors will consider your comments a prerequisite.

**Reviewer #2:**

**Comment:** The manuscript is well written, the research study is of good quality, and the topic is of high importance in covering a pressing issue. Publications in this area are necessary. The researchers' access to Gaza could be improved, making the study very valuable.

**Response:** Thank you, we are glad to hear.

Some minor writing mistakes need to be corrected

**Comment:** On page 10, I suggest adding some information on the history of the blockade on Gaza.

**Response:** Added as suggested; see lines 422-439.

**Comment:** On page 12, the word "report" is repeated repeatedly, making this sentence redundant: "the report of the Global Nutrition Cluster (GNC) Report."

**Response:** Corrected; see lines 503-504.

**Comment:** Page 12 GNS should be GNC

**Response:** Corrected; see lines 505,509.

**Comment:** Page 15 Middle GA should be clarified

**Response:** Clarified as Middle Gaza; see line 654.

**Reviewer #3:**

**Comment:** General Comments: This paper addresses a highly relevant and urgent issue by examining hunger levels among Gaza residents after the military escalation on October 7th. The study aims to provide population-based data that could be valuable for informing policymakers and humanitarian organizations on addressing hunger and food insecurity.

**Detailed Comments:**

**Title:
Comment:** The title contains the word "Hunger, " which is redundant. A clearer, more concise alternative is "Catastrophic Hunger in Gaza: A Population-Based Study of Unprecedented Food Insecurity Post-October 7^th^".

**Response:** The title has been corrected and improved as per the suggestions, and famine and hunger are now included. The title becomes Catastrophic Famine in Gaza: Unprecedented Levels of Hunger Post-October 7^th^. A Real Population-Based Study from the Gaza Strip**.** Corrected; see the title on page 1, lines 1-2.

**Abstract:
Comment:** Background: The start is strong, but the ending mentions informing policymakers about strategies to mitigate hunger, which needs to be discussed in the paper. The purpose should instead emphasize providing insights into hunger levels and food insecurity rather than strategy.

**Response:** Corrected as suggested, please see lines 19-22, 41-44, 118-120, 380, 603, 606-613.

**Comment:** Methods: Sociodemographic data should be included in this section for completeness.

**Response:** Added, see lines 28-34.

**Introduction:
Comment:** The background of the Gaza crisis is well covered, but the section should include the most recent reports on hunger and malnutrition in Gaza.

**Response:** The most recent report of the UNGPH on October 2024 is added. See line 62-63.

**Comment:** Definitions of key terms like hunger, famine, starvation, and food security should be included to distinguish these closely related concepts.

**Response:** Added, se lines 76-94.

**Comment:** The introduction describes the Household Hunger Scale (HHS) in detail but merely mentions the Household Food Insecurity Access Scale (HFIAS) and the Household Food Security Survey Module (HFSSM). More balanced coverage is needed.

**Response:** The HHS text is significantly reduced to be comparable to the description of the other two tools, and the HFIAS and HFSSM are further described. A more balanced description can be found in lines 95-117.

**Comment:** It’s important to clarify that the study's purpose is to inform policymakers rather than to create strategies that align with the abstract.

**Response:** Corrected as suggested, please see lines 19-22, 41-44, 118-120, 380, 603, 606-613.

**Materials and Methods:**

**Comment:** This is a cross-sectional study conducted in the Gaza Strip from May to July 2024 during the ongoing war in the region. There is no clear description of how data were collected or whether the data collectors were trained.

**Response:** Description is added; see lines 131-135.

**Comment:** The method of data collection (e.g., paper-based, Google Docs) should be specified.

How the questionnaire was administered (online or in-person) needs to be clarified, though later in the results section, it is mentioned that households were interviewed.

**Response:** The same as before, the description has been added; see lines 131-135.

**Comment:** Participant eligibility criteria, informed consent processes, and an explanation of household hunger categories are absent.

**Response:** Informed consent is already there; see lines 137 and 145. Inclusion criteria are found in lines 141-145.

**Comment:** The explanation of the HFIAS tool could be made easier to understand through a table or appendix.

**Response:** The tool's explanation is transferred to the footnote of Table 4 for the HFIAS and removed from the main text. See Table 4, page 26. The reader is referred to the main reference [18].

**Comment:** The full questionnaire should be included in the appendix, and details about its development, validation, and scope (e.g., anthropometrics and sociodemographics) should be clearly outlined. It wasn’t clear how the questionnaire was built until we discovered that it combined the three scales with other questions.

**Response:** Added, see lines 152-157, 159-166.

**Comment:** More details are needed on how anthropometric data were collected and organized.

**Response:** The anthropometrics were self-reported, not measured (see the subheading, line 169). The validity and reliability of the self-reported anthropometric measurements are well documented in many published articles and systematically reviewed in one published article. Fayyaz et al. [26]; see lines 186-193.

**Comment:** The translation of assessment tools should mention who performed the translations and their qualifications.

**Response:** Added, see lines 152-154.

**Comment:** After viewing the data, a major flaw is the inconsistency in the data categories and geographical or current address names, which should be standardized to allow for proper analysis.

**Response:** The Gaza Strip is divided into three large governorates (prior to the recent Israeli reoccupation): the northern governorates (North Gaza, Gaza City), the middle (Deir Al Balah), and the southern (Khan Younis, Rafah). This governmental classification is consistent throughout the manuscript; see lines 130-131, 218-220, 325-330, 357-360, and 514.

**Results:**
**Comment:** Some data, such as economic status, should have been mentioned in the methods section but appear here for the first time. How was economic status categorized? What are the boundaries, and according to what?

**Response:** The respondents self-reported their economic status before the war; see lines 32, 162, 338, 366, and Table 1.

**Comment:** The presentation of results would benefit from visual aids like graphs or figures, particularly for illustrating shifts in BMI and weight loss.

**Response:** Done, added, see page 28.

**Comment:** The conclusion contradicts the results by stating that camp dwellers have higher hunger severity, which is not reflected in the results.

**Response:** This was described in Table 7 (which now becomes Supplementary Table 2), the question on Address before the war (City house/Home or Camp) and its relation to the level of household hunger.

**Comment:** When discussing the governorates, it is unclear whether the household location refers to where they are currently due to continuous displacement or where they originally came from.

**Response:** It was where they originally came from.

**Comment:** The phrase "with particular attention toward the three or more months by about one-third of the respondents" is unclear. How was this determined? Was it part of the questionnaire or a note from interviewers?

**Response:** The “three months” is mentioned in questions 5, 10, and 17 of the HFSSM (Table 3). The confusing statement has been rephrased for better clarity. See lines 271-273.

**Comment:** Results should be summarized clearly before diving into further detail.

**Response:** Done for the main five Tables (1-5) (the rest of Tables 6-8 are separated as Supplementary Tables 1-3, as indicated by one of the reviewers), see lines 215-218 (Table 1), 240-242 (Table 2), 260-265 (Table 3), 280-283 (Table 4), and 294-297 (Table 5).

**Discussion:
Comment:** The discussion is too lengthy and redundant in parts, especially when detailing the situation in Gaza before the war, which is not particularly related to the research aim. It should focus more on the study’s findings about hunger and food insecurity, backed by current evidence.

**Response:** The discussion on the nutritional status before the war is removed, and current evidence is added in this context. However, due to the extra new requirements suggested by the respected reviewers, the discussion becomes lengthier. More than ten new references were added and used in the discussion part.

**Comment:** The omission of study limitations, such as challenges faced during data collection in a conflict zone (e.g., safety, displacement, internet access, movement), especially in Northern Gaza, is significant. For example, the Rafah invasion and evacuation occurred at the beginning of the research period. How did this affect the research?

**Response:** Research limitations are expanded, and extra factors are added; see lines 615-637. Regarding the Rafah invasion and people's evacuation, people reached out in their newly arrived places, in their tents of camps, empty schools, or partially damaged houses, and were interviewed after being evacuated from Rafah.

**Comment:** More references are needed to strengthen some statements, and comparisons to similar studies would enhance the discussion.

**Response:** A comparison with relevant studies of famine and food insecurity amid military conflicts in Ethiopia and Nigeria has been added; see lines 398-400.

**Comment:** Non-participation challenges due to internet and war-related issues, such as the inability to reach people in tents either in the field or online, should be highlighted.

**Response:** Added, see lines 625-637.

**Comment:** The discussion should emphasize how the study’s results can inform humanitarian organizations in addressing hunger.

**Response:** Added; see lines 580-586, 613-614.

**Comment:** Contradictions arise when Southern Gaza is shown to have higher hunger rates than Northern Gaza, which contradicts previous reports of more severe conditions in the north. Please support your results and expand on this topic.

**Response:** Contradictions arise when Southern Gaza reports higher hunger rates than Northern Gaza, despite earlier assessments indicating more severe conditions in the north. This discrepancy can be understood through the geographical context and the original locations of displaced individuals. Historically, residents of Northern Gaza, especially in cities like Gaza City, have had a higher economic status and better access to income. This economic advantage has enabled many to retain savings, gold, or financial assistance, enhancing their ability to cope with crises and resulting in lower hunger prevalence. In contrast, individuals in Southern Gaza often lack similar resources, making them more susceptible to food insecurity. Additionally, many displaced people from Northern Gaza relocated to the Middle and Central areas during our data collection, further influencing hunger dynamics. These economic factors are crucial in understanding the varying hunger rates across different regions of Gaza; see lines 521-530.

**Conclusion:
Comment:** The conclusion needs to be aligned with the rest of the paper, summarizing and validating the findings. It should refrain from introducing new elements, like the cessation of hostilities, for the first time here.

**Response:** Please see lines 650-658.

**References:
Comment:** Some references, such as those for ICRC protocols and the Global Nutrition Cluster, need corrections and consistency.

**Response:** Corrected, thank you. Please check the reference numbers 40 and 41.

**Images/Tables (if any):**

**Comment:** Tables 6-8 could be included as supplementary files, with only the significant p-values mentioned in the main text.

**Response:** Done. Tables 6-8 are separated and included as supplementary files (Supplementary Table 1-2).

**Comment:** Figures representing weight changes and hunger severity would improve clarity.
**Response:** Changes in body weight and BMI are depicted in Figure 1, page 28. Hunger severity is a single value, not pre-post-war, and is reported in the tables for each assessment tool.

**Comment:** The overall language is clear and acceptable, though minor improvements could enhance readability.

**Response:** Thank you. The manuscript underwent comprehensive editing and linguistic revision. I hope all the minors are resolved.

**Reviewer #4:**

**Research Design and Rationale:**

**Comment:** The abstract clearly states that the findings aim to inform policymakers and humanitarian organizations, but could you clarify how they are specifically intended to inform policy or interventions in Gaza?

**Response**: Thank you for your insightful comment regarding the applicability of our findings to policymakers and humanitarian organizations. Due to the limited word count in the abstract, no further details can be added. This can be found more clearly in the text; see lines 41-43, 118-120, 607-614.

**Comment:** Are there any particular groups or strategies you envision targeting with the findings?

**Response:** Thank you for your question. Our findings will specifically target vulnerable populations, such as low-income families, pregnant and lactating women, older adults, and children while informing local NGOs, international humanitarian agencies, and policymakers to develop tailored strategies that address the underlying issues of food insecurity in Gaza; see lines 659-662.

**Justification of Study Location.**

**Comment:** Why did you choose to conduct this study in Gaza, specifically during this period of conflict?

**Response:** Thank you for your question. We are conducting this study in Gaza during Israeli military attacks to assess the rising food insecurity and famine resulting from the humanitarian crisis. This context enables us to gather timely data to inform humanitarian responses and policy interventions aimed at alleviating suffering and addressing urgent needs. Our research also aimed to provide actionable insights to shape both immediate and long-term support strategies for those affected by the conflict; see lines 379-389.

**Comment:** Given the highly dynamic situation in Gaza, could you elaborate on how the timing of the study (May to July 2024) may have influenced the findings?

**Response:** Our study from May to July 2024, conducted after 7-9 months of Israeli military attacks, aimed to capture the evolving humanitarian crisis in Gaza. This timeframe allowed us to assess the immediate impact on food security, hunger, and famine, reflecting the acute challenges faced, such as supply disruptions, economic hardship, and displacement. Local and international responses also influenced the effectiveness of support for affected communities. We recognize that our findings represent a snapshot of a dynamic situation, emphasizing the need for ongoing research to monitor changes over time and inform immediate humanitarian efforts; see lines 383-389.

**Previous Literature:**

**Comment:** How does your study compare to existing research on food insecurity in conflict zones, especially in Gaza? Are there any notable discrepancies with previous studies that report lower levels of food insecurity, and how do you explain these differences?

**Response:** Thank you for your question. Our study enhances existing research on food insecurity in conflict zones, particularly Gaza, by providing updated data amid the current humanitarian crisis. While previous studies reported varying food insecurity levels, we observed significantly higher rates during our research period, which was attributed to several factors. The timing of our study aligns with a particularly intense phase of Israeli attacks, exacerbating vulnerabilities, increasing displacement, disrupting food supply chains, and heightening economic hardship for many conditions that earlier studies may not have fully captured, see lines 453-459.

**Sampling and Data Collection:**

**Sampling Strategy:**

**Comment:** The study surveyed 1209 households out of an initial 1503. Could you provide more details about the reasons behind excluding 294 households? Was this due to missing data, or were there other factors that may have affected the final sample?

**Response:** Thank you for your question. The final analysis excluded 294 households primarily due to missing or incomplete data, which were essential for accurate analysis. Some households could not provide the necessary information, while others faced logistical challenges, such as access issues caused by ongoing Israeli military attacks or displacement. Additionally, some respondents chose not to complete their participation due to safety concerns or mistrust of the survey process; see lines 209-214.

**Demographic Representation:**

**Comment:** You mention that the mean age of participants is 38, with 53.5% being female. Were these demographics reflective of the overall Gaza population? Did you find any significant gender or age-based differences in food insecurity and hunger levels?

**Response:** Thank you for your question. The demographics of our study reflect the overall population in Gaza, with a significant proportion of adults in their mid-to-late thirties and a fairly balanced sex ratio. Our analysis revealed notable disparities in food insecurity based on sex, with female-headed households experiencing higher levels of food insecurity than male-headed households, highlighting the unique challenges women face in accessing resources during the ongoing Israeli military attacks. Age-based differences were also evident, as participants under 30 reported higher food insecurity levels compared to older age groups, likely due to limited employment opportunities and increased reliance on family support. Overall, our findings indicate that demographics significantly influence food insecurity experiences in Gaza, emphasizing the importance of these interactions for informing targeted interventions and assistance strategies; see lines 231-239.

Data Collection in a Conflict Zone:

**Comment:** Given the ongoing conflict, were there any particular challenges or limitations in collecting data in Gaza? For example, how did the security situation impact the study’s logistics or participants' willingness to engage?

**Response:** Thank you for your question. While we collected self-reported anthropometric data, including weight changes, we acknowledge that stress and trauma from ongoing Israeli military attacks may affect reporting accuracy. Participants may need help to provide precise information or recall changes due to emotional impacts, which should be considered when interpreting our findings. Additional limitations include challenges in accessing participants due to security concerns, logistical difficulties, and potential selection bias from displacement and restricted movement. Disrupted infrastructure and communication in conflict zones may hinder accurate and timely data collection. Recall bias is also a concern, as stress may affect participants’ abilities to remember their food insecurity experiences accurately. The rapidly evolving situation in Gaza can lead to fluctuating food insecurity levels, complicating consistent data capture. Political and ethical dilemmas regarding sensitive data reporting may also influence findings, while limited funding could restrict the study's scope; see lines 641-644.

Self-Reported Anthropometric Data:

**Comment:** You collected self-reported anthropometric data, such as weight changes. How confident are you in the accuracy of these self-reports, considering the stress and trauma participants may be experiencing? Please insert this as a limitation.

**Response:** While we collected self-reported anthropometric data, including weight changes, we acknowledge that stress and trauma from ongoing Israeli military attacks may affect reporting accuracy. Participants may struggle to provide precise information or recall changes due to emotional impacts, which should be considered when interpreting our findings, see lines 638-643.

**Interpretation of Results:**

**Hunger and Weight Loss:**

**Comment:** The average weight loss (from 74.6 kg to 64.1 kg) is striking. What do you believe are the key contributing factors to this weight loss beyond food insecurity, such as stress, healthcare access, or psychological impacts?

**Response:** Thank you for observing the significant average weight loss from 74.6 kg to 64.1 kg among participants. As per our results, the war has caused considerable weight loss, with the average weight decreasing from 74.6 kg before the conflict to 64.1 kg. While food insecurity is a primary factor, other key contributors include stress and trauma from ongoing attacks, which can alter eating habits and lead to anxiety and depression[1, 2]. Limited access to healthcare exacerbates health issues, potentially causing further weight loss[3]. Additionally, the psychological impacts of living in a conflict zone can disrupt eating patterns, causing emotional eating or loss of appetite [4]. The chaotic environment also hinders regular food preparation and consumption. In summary, the interplay of food insecurity, stress, healthcare access, and psychological effects significantly contributes to the weight loss observed; see lines 410-418.

**Statistical Impact of Confounding Variables:**

**Comment:** The abstract mentions that factors like displacement, age, economic status, and education significantly impacted hunger severity. Could you provide more details on the extent to which these variables influenced your findings? Were there any surprising or unexpected relationships?

**Response:** Thank you for your question regarding the factors influencing hunger severity in our study. Our analysis showed that displacement, age, economic status, and education significantly impact hunger levels in distinct ways. Displacement was a key factor, with displaced individuals reporting higher food insecurity due to loss of livelihoods and support networks. Younger individuals, particularly those under 30, experienced greater food insecurity, likely due to limited employment opportunities. Economic status emerged as a major determinant, with lower-income households facing severe hunger amid the ongoing Israeli military attacks, which have disrupted job availability and income stability. Interestingly, education had a complex relationship with hunger severity. While higher education typically correlates with better economic opportunities, individuals with some post-secondary education faced unique challenges in securing jobs during the conflict, leading to unexpected food insecurity levels. Our findings revealed that economic status significantly influenced hunger severity, more so than anticipated, highlighting the need for targeted economic interventions. In summary, these interconnected variables distinctly shape food insecurity experiences in Gaza, providing crucial insights for effective intervention strategies; see lines 441-452.

**Policy and Humanitarian Implications:**

**Recommendations for Action:**

**Comment:** Based on your findings, what specific recommendations would you propose to humanitarian organizations operating in Gaza to mitigate hunger and improve food security? Are there any quick-win interventions that could be implemented in the short term?

**Response:** Thank you for your question regarding recommendations for humanitarian organizations to mitigate hunger and improve food security in Gaza. Based on our findings, we propose specific recommendations, including long-term strategies and quick-win interventions. First, targeted food assistance programs should be established to address the immediate needs of vulnerable populations, particularly displaced families and low-income households. Cash or food vouchers can empower families to choose based on their dietary needs and stimulate local markets. Second, nutrition education programs should be implemented to help families make informed choices about food preparation and healthy eating during periods of limited food access. For long-term strategies, invest in livelihood support initiatives to create job opportunities for younger individuals and women through training programs or small business support aligned with local market needs. Improving coordination among humanitarian organizations is vital for streamlining efforts and enhancing intervention effectiveness. Quick-win interventions can include community food distribution initiatives targeting neighborhoods most affected by food insecurity and community gardening projects to promote food sovereignty. By prioritizing these recommendations, humanitarian organizations can significantly improve food security and mitigate hunger in Gaza. Ultimately, ending the Israeli occupation and ensuring Palestinians' rights to live with dignity and freedom are essential for resolving these issues; see lines 573-586.

**Long-term Policy Recommendations:**

**Comment:** Beyond immediate relief efforts, what longer-term policy changes do you think are necessary to address food insecurity in Gaza, particularly considering the ongoing conflict and blockade? Are there any impact on SDGs? Please indicate the importance of your study, which shows the uselessness of international humanitarian rights and the SDGs.

**Response:** Thank you for your question regarding the longer-term policy changes needed to address food insecurity in Gaza. Our study underscores the urgent need for these policy changes. It highlights the inadequacy of current international humanitarian responses, which need to align better with the Sustainable Development Goals (SDGs), particularly Goal 2 (Zero Hunger) and Goal 1 (No Poverty). While humanitarian efforts provide crucial immediate relief, they fall short of addressing systemic issues stemming from ongoing conflict and socioeconomic instability. Integrating humanitarian action with political solutions is necessary to ensure that international frameworks and rights extend beyond mere formal declarations. With addressing these deeper structural issues, the objectives of the SDGs will be met, and communities in Gaza will continue to suffer from severe food insecurity and human rights violations. By advocating for these essential policy changes and identifying gaps in current humanitarian approaches, our study aims to inform more effective and sustainable interventions aligned with international human rights and development frameworks; see lines 678-695.

1.	Bremner JD, Moazzami K, Wittbrodt MT, Nye JA, Lima BB, Gillespie CF, et al. Diet, Stress and Mental Health. Nutrients. 2020;12(8). Epub 2020/08/23. doi: 10.3390/nu12082428. PubMed PMID: 32823562; PubMed Central PMCID: PMCPMC7468813.

2.	Celik Erden S, Karakus Yilmaz B, Kozaci N, Uygur AB, Yigit Y, Karakus K, et al. The Relationship Between Depression, Anxiety, and Stress Levels and Eating Behavior in Emergency Service Workers. Cureus. 2023;15(2):e35504. Epub 2023/04/04. doi: 10.7759/cureus.35504. PubMed PMID: 37007378; PubMed Central PMCID: PMCPMC10058381.

3.	Washington TB, Johnson VR, Kendrick K, Ibrahim AA, Tu L, Sun K, et al. Disparities in Access and Quality of Obesity Care. Gastroenterol Clin North Am. 2023;52(2):429-41. Epub 2023/05/18. doi: 10.1016/j.gtc.2023.02.003. PubMed PMID: 37197884; PubMed Central PMCID: PMCPMC10198472.

4.	Aksonova O, Slivar D, Torianik D, Gubsky S. The food security and the eating behavior of the civilian population during the military conflict in Ukraine: a preliminary research. Nutrition & Food Science. 2024;54(1):251-63.
