# Supplementary material for: Catastrophic famine in Gaza: Unprecedented levels of hunger post-October 7th. A real population-based study from the Gaza Strip
Source: PLoS One. 2025 May 28;20(5):e0309854. doi: 10.1371/journal.pone.0309854 (PMC12118885; doi:10.1371/journal.pone.0309854)
Supplement: Attachment — Submitted filename: Rebuttal PLOSONE R2 1222025 (AutoRecovered).docx [file pone.0309854.s008.docx]

Editor, PLOS ONE

Public Library of Science

***Subject: Revised Manuscript Submission: Catastrophic Famine in Gaza: Unprecedented Levels of Hunger Post-October 7th. A Real Population-Based Study from the Gaza Strip***

Dear Editor,

Thank you for the opportunity to revise and resubmit our manuscript titled "*Catastrophic Famine in Gaza: Unprecedented Levels of Hunger Post-October 7th. A Real Population-Based Study from the Gaza Strip*." We appreciate the time and consideration given to our work by the editors and reviewers.

We have carefully addressed all the comments and suggestions raised during the review process. The revised manuscript is significantly strengthened as a result of the reviewers' insightful feedback. We have incorporated all of the requested changes, and we believe that the manuscript now meets the high standards of PLOS ONE.

We want to express our sincere gratitude to the reviewers for their constructive criticism and helpful suggestions. Their feedback has been invaluable in improving the quality, readability, and clarity of our manuscript.

This study provides crucial insights into the devastating impact of the recent conflict on food security and hunger in Gaza. The findings highlight the urgent need for humanitarian assistance and intervention to address the ongoing crisis. Our work will contribute to raising awareness of this critical issue and inform efforts to alleviate the suffering of the people in Gaza.

Thank you for your continued consideration of our manuscript.

We look forward to your final decision.

Sincerely,

MoezAlIslam,

[moezalislma@gmail.com](mailto:moezalislma@gmail.com)

On behalf of all authors

**Editors’ comments:**

- **Comment 1:** Provide additional appropriate references to support lines 424-434, "The current...food availability."
- **Response:** Added, see references 29-32. Lines 486, 492, 495.
- **Comment 2:** Remove lines 402-403, "Our finding indicates...Israeli policies", the end of line 425, "In retaliation for this political choice," lines 469-470 "The current...war laws," and 484-485 "The deliberate...humanitarian catastrophe", and any other content that speculates on the motivations of any person or institution.
- **Response:** Done, removed.
- **Comment 3:** Remove lines 532-565, "The humanitarian crisis...population in Gaza," and any other content that analyzes the legality or legitimacy of any person or institution.
- **Response:** Done, removed.
- **Comment 4:** Remove lines 665-666, "The international community...Palestinian people" and 668-670, "Additionally, advocating for...in the region", and ensure in particular that the Conclusions section is restricted to interpretations that are directly supported by the data presented in the results of the manuscript.
- **Response:** Done, removed.
- **Comment 5:** Remove the following keywords and any other language that might be perceived as inflammatory, from your submission: Zionism, War criminals
- **Response:** Done, removed.

**Reviewer’s comments:**

**Specific Comments**

**Abstract**

- **Comment 2:** The abstract provides a concise summary but could better emphasize key findings and their implications.
- **Response:** Done, please see the abstract. Key findings are more emphasized.
- **Comment 2:** Revise the sentence on informing policymakers (lines 19–22) to focus on actionable insights rather than general aims.
- **Response:** Done, kindly see lines 22.

**Introduction**

- **Comment 2:** While the introduction offers comprehensive background information, it could be more concise, especially when discussing the historical context of food insecurity in Gaza.
- **Response:** The historical context of food insecurity in Gaza is reduced to be more concise; see lines 60-68.
- **Comment 2:** Definitions of key terms like hunger, food insecurity, and famine (lines 76–94) are helpful but should be condensed to improve clarity and avoid redundancy.
- **Response:** Done, please see lines 76-81.

**Methods**

- **Comment 2:** To enhance the reliability and validity of the methods, details on inclusion criteria (lines 141–145) and the translation process (lines 152–154) should be expanded.
- **Response:** Done, see lines 145-153, 160-172.
- **Comment 2:** Elaborate on the training process for data collectors (lines 131–135) to strengthen the methodological rigor.
- **Response:** Done, see lines 124-138.

**Results**

- **Comment 2:** The results are detailed, but incorporating visual aids such as graphs or charts would enhance clarity and engagement, particularly for shifts in BMI and hunger prevalence.
- **Response:** Added, please see Figures 1-3, pages 30-32.
- **Comment 2:** The categorization and impact of economic status on food insecurity (line 162) should be further elaborated.
- **Response:** Added, please see lines 185-202.
- **Comment 2:** Provide clearer categorization and contextual analysis of displacement data (line 130).
- **Response:** Added, see lines 203-214.

**Discussion**

- **Comment 2:** The discussion is thorough but occasionally redundant. Focus more on how the findings can directly inform policy and humanitarian interventions.
- **Response:** Please find in lines 604-618.
- **Comment 2:** The study limitations (lines 615–637) are well-addressed but should also include potential biases related to self-reported data.
- **Response:** Added, see lines 692-700.

**Conclusion**

- **Comment 2:** The conclusion is consistent with the findings but introduces new elements, such as the cessation of hostilities (line 650), which should be integrated earlier in the discussion.
- **Response:** Added, please see lines 602.

**References**

- **Comment 2:** Ensure consistency and accuracy in citations, particularly for references such as ICRC protocols (lines 40–41).
- **Response:** The paragraph including ICRC is removed based on one of the reviewer’s suggestions.

**Figures and Tables**

- **Comment 2:** Incorporate visual representations for key findings, such as weight loss and hunger severity, to improve the impact of the manuscript (e.g., BMI changes on Page 14).
- **Response:** Added, please see Figures 1-3, pages 30-32.

**Page-Specific Comments**

- **Comment 2: Page 5, Line 62**: Incorporate more recent reports on hunger to provide up-to-date context.
- **Response:** Not clear; page 5 includes lines 143-173, while line 62 is present on page 2. However, to meet this requirement, recent reports on hunger amid the armed conflicts in Sudan, Ethiopia, and Nigeria are added to provide up-to-date context. See lines 60-62.
- **Comment 2: Page 10, Line 162**: Clarify how economic status categories were defined and determined.
- **Response:** Added, please see lines 185-202.
- **Comment 2: Page 14, Table 4**: Simplify the table and key trends in the narrative.
- **Response:** Table 4 is presented on page 10, not 14. The table cannot be simplified more as it reflects the nine components of the assessment tool of HFIAS. In the narrative, key trends are presented; see lines 334-338.
- **Comment 2: Page 18, Line 453**: To contextualize the findings, compare them with similar studies on food insecurity in other conflict zones.
- **Response:** Added, see lines 462-464.
